# Varus stress MRI in the refined assessment of the posterolateral corner of the knee joint

**DOI:** 10.1038/s41598-022-15787-2

**Published:** 2022-07-13

**Authors:** Malin Ciba, Eva-Maria Winkelmeyer, Justus Schock, Simon Westfechtel, Teresa Nolte, Matthias Knobe, Andreas Prescher, Christiane Kuhl, Daniel Truhn, Sven Nebelung

**Affiliations:** 1 Department of Diagnostic and Interventional Radiology, Aachen University Hospital, Pauwels Street 30, 52074 Aachen, Germany; 2 Medical Faculty, University of Zurich, Zurich, Switzerland; 3 Medical Faculty, RWTH Aachen, Aachen, Germany; 4 Institute of Molecular and Cellular Anatomy, RWTH Aachen University, 52074 Aachen, Germany

**Keywords:** Tissues, Experimental models of disease, Translational research, Diagnostic markers, Magnetic resonance imaging, Three-dimensional imaging

## Abstract

Magnetic resonance imaging (MRI) is commonly used to assess traumatic and non-traumatic conditions of the knee. Due to its complex and variable anatomy, the posterolateral corner (PLC)—often referred to as the joint’s dark side—remains diagnostically challenging. We aimed to render the diagnostic evaluation of the PLC more functional by combining MRI, varus loading, and image post-processing in a model of graded PLC injury that used sequential transections of the lateral collateral ligament, popliteus tendon, popliteofibular ligament, and anterior cruciate ligament. Ten human cadaveric knee joint specimens underwent imaging in each condition as above, and both unloaded and loaded using an MR-compatible device that standardized loading (of 147 N) and position (at 30° flexion). Following manual segmentation, 3D joint models were used to computationally measure lateral joint space opening for each specimen, configuration, and condition, while manual measurements provided the reference standard. With more extensive ligament deficiency and loading, lateral joint spaces increased significantly. In conclusion, varus stress MRI allows comprehensive PLC evaluation concerning structural integrity and associated functional capacity. Beyond providing normative values of lateral compartment opening, this study has potential implications for diagnostic and surgical decision-making and treatment monitoring in PLC injuries.

## Introduction

The posterolateral corner (PLC) used to be referred to as the *dark side* of the knee due to limited knowledge of its structure and function^1^. Although common understanding of its anatomic and biomechanical intricacies has improved over the past decades^2–5^, PLC injuries are still largely underreported^6,7^. Though isolated PLC injuries are rare, their incidence is substantial in patients with multiple knee ligament injuries. LaPrade et al. reported PLC injuries in 16% of patients with acute ligament injuries^6^, while Becker et al. reported PLC injuries in 77% of multi-ligamentous knee injuries^8^. Delayed and missed treatment predisposes the joint to chronic instability, persistent pain, failure of cruciate ligament reconstructions, and, eventually, osteoarthritis^9,10^. Pacheco et al. found that 72% of patients with PLC injuries were not diagnosed correctly at initial presentation, which delayed treatment by a mean duration of 30 months after injury^11^. These deficits are of particular relevance because timely diagnosis and treatment are essential prerequisites for favorable long-term outcomes^12^. Current diagnostic strategies are centered on patient history, physical examination, and imaging findings^9^. PLC injuries usually occur in combination with other knee joint injuries^6,8,11,13^, and, therefore, physical findings of PLC injuries are often equivocal because of concomitant injuries^9,10^. Conventional and stress radiographs may be used, in particular, to quantify lateral compartment opening^10,14,15^; yet radiographic assessment is inherently limited because it lacks direct visualization of the injured structure, and specificity, reliability, and reproducibility are low^16,17^.

Due to its excellent soft-tissue contrast, magnetic resonance imaging (MRI) is the state-of-the-art imaging modality of contemporary musculoskeletal imaging and is indispensable to confirm (or rule out) PLC injuries^12,18^. After an acute injury, the larger structures of the PLC, i.e., the lateral collateral ligament (LCL) and the popliteus tendon (PT), can be reliably identified and assessed in terms of structural integrity^19^. Yet, due to their small size and oblique orientation, other structures such as the popliteofibular ligament (PFL) are quite challenging to evaluate^13,19–23^, in particular in the presence of concomitant injuries, soft-tissue edema, or joint effusion^21^. Due to their variable morphology chronic injuries with variable signs of tissue healing and scarring are challenging, too, and MRI may be of limited use only^24^. Moreover, current MRI protocols of the knee are usually acquired with the patient supine (and the joint unloaded), which is unfunctional and unlike the joint’s physiologic configuration.

Given these shortcomings, this study’s objective was to render the assessment of the PLC more functional by implementing varus stress MRI, i.e., the combination of MRI with standardized varus loading. To this end, we brought together clinical MRI sequences, pressure-controlled varus loading, advanced image post-processing, and an in-situ model of graded PLC injuries to study the effects of sequential PLC injuries on lateral joint stability (for which lateral compartment opening under varus stress served as the imaging surrogate). We hypothesized that lateral compartment opening under varus stress is related (i) to the extent of PCL injury and (ii) the underlying measurement methodology.

## Results

All 10 human knee joint specimens underwent consecutive MRI in two configurations (i.e., unloaded and loaded) and in five conditions (i.e., intact; LCL deficient; LCL and PT deficient; LCL, PT, and PFL deficient; and LCL, PT, PFL, and anterior cruciate ligament [ACL] deficient).

**Figure 1:**
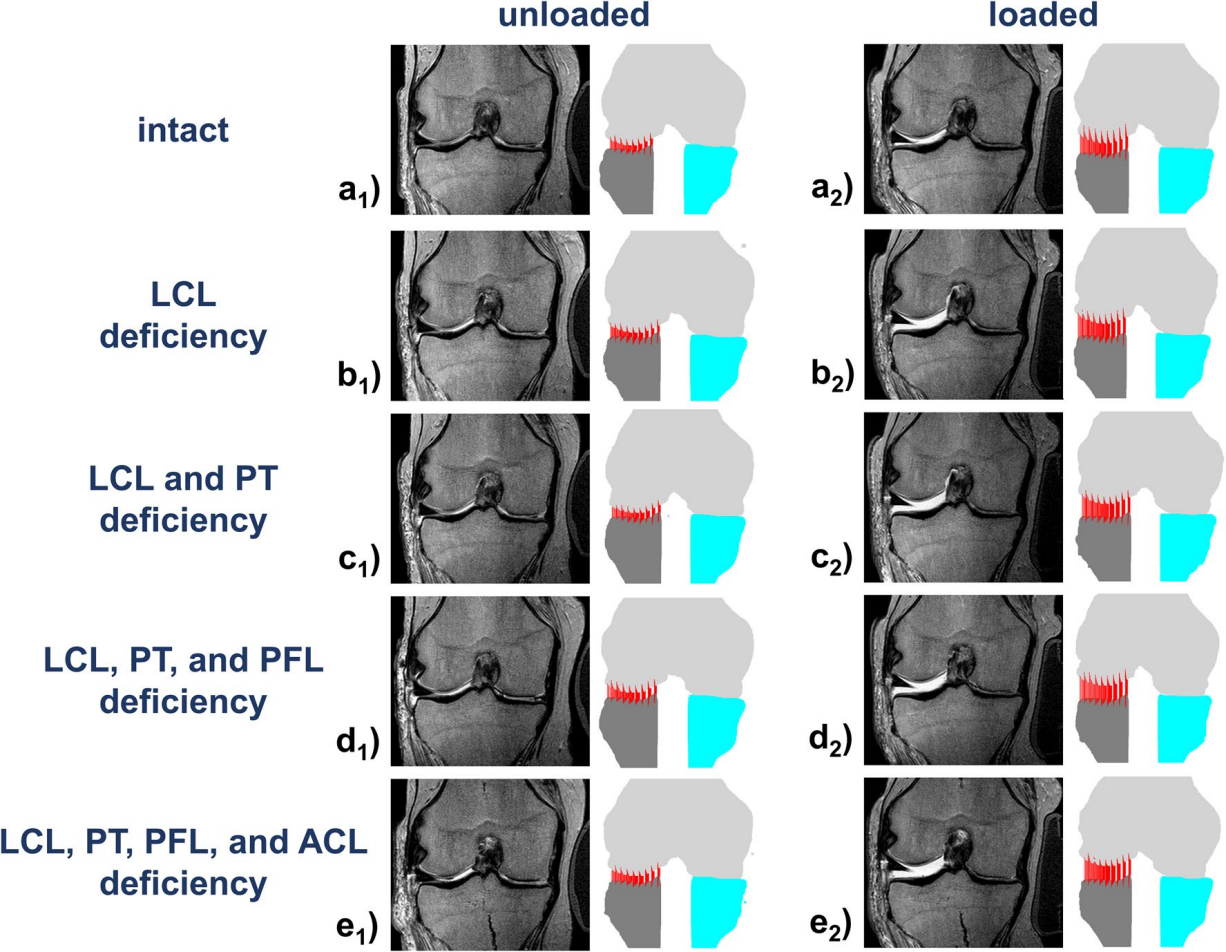
Visualization of the femorotibial joint as a function of varus loading and extent of injury to the posterolateral corner. Mid-coronal T2-weighted images (left) and corresponding visualizations of the 3D joint model (right) of a representative specimen in the loaded (**a**_**1**_–**e**_**1**_) and unloaded (**a**_**2**_–**e**_**2**_) configurations. Conditions are intact (**a**) and after surgical transections of the lateral collateral ligament (LCL, **b**), popliteus tendon (PT, **c**), popliteofibular ligament (PFL, **d**), and anterior cruciate ligament (ACL, **e**). Color codes are light grey for the femur, turquoise for the medial tibia, and dark grey for the lateral tibia. Vertical red lines indicate individual subchondral cortical distances used for computational measurements.

**Figure 2:**
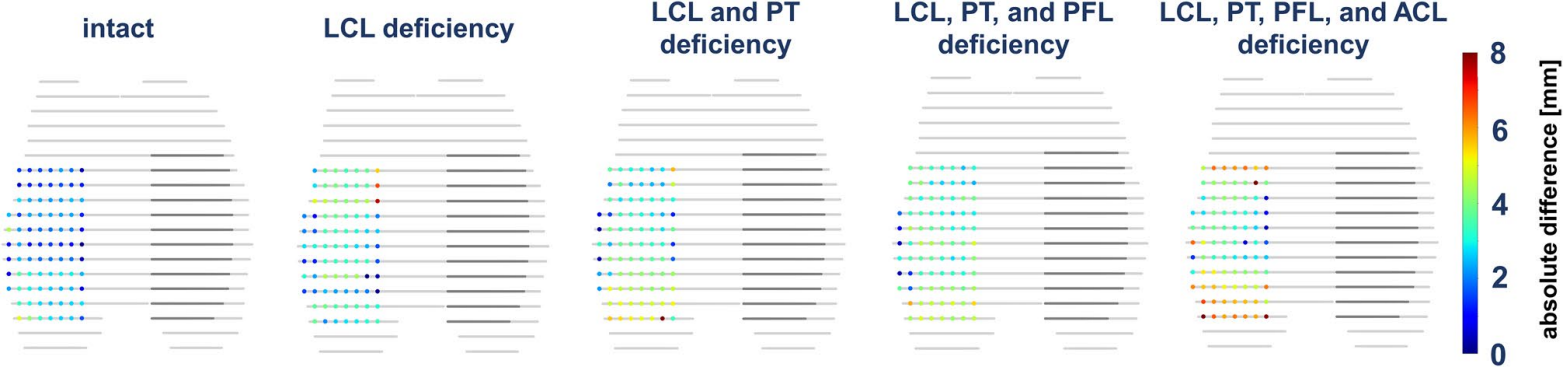
Spatially resolved heat maps of lateral compartmental opening under varus loading as a function of the extent of injury to the posterolateral corner. For each grid point of the lateral compartment, the absolute differences in subchondral cortical distances were quantified between each condition's unloaded and loaded configurations. Displayed are axial reconstructions of the 3D models and the mean absolute differences across all specimens. Color-coded scale on the right indicates absolute differences (mm). *LCL* Lateral collateral ligament, *PT* popliteal tendon, *PFL* popliteofibular ligament, *ACL* anterior cruciate ligament.

Qualitatively, the lateral compartment hardly changed in the unloaded configurations, even in the most extensively injured condition. Yet, once loaded, the lateral compartment increasingly opened as a function of PLC injury (Fig. 1). Spatially resolved analysis of the loading-induced changes across the lateral compartment indicated that the largest amount of compartmental opening was present in the posterior and—to a lesser extent—anterior regions of the lateral compartment (Fig. 2).

Quantitatively, absolute changes in the subchondral cortical distances (SCDs) at specific locations as well as their overall means were reflective of these observations. With the increasing extent of injury to the PLC and loading, SCD values were—by and large—significantly increased, irrespective of the underlying measurement method (Table 1). Post-hoc results indicated significant pair-wise post-hoc differences primarily for the loaded configurations, while for the unloaded configurations, SCD values were largely unaltered and only marginally increased with the increasing extent of injury (Supplementary Table 1). Consequently, between the intact unloaded and completely deficient (i.e., PLC- and ACL-deficient) unloaded configurations, overall mean SCD values differed by only 0.9 mm (manual measurements) and 0.8 mm (computational measurements), respectively.

**Table 1:** Manually and computationally measured subchondral cortical distances (SCDs) as a function of loading configuration and joint condition. Manual reference measurements of SCDs were performed by two readers at six locations along the mediolateral (SCD_ml_) and anteroposterior (SCD_ap_) dimensions, respectively, and pooled data are indicated. Manually measured SCDs across the six locations were averaged (SCD_manmean_). Computational measurements were performed using a grid of locations covering the entire lateral joint compartment, while, for comparison, SCD values for those grid points closest to the manual measurement locations (SCD_ml_, SCD_ap_) are indicated. Computationally measured SCDs across all grid points of the entire lateral joint compartment were averaged, too (SCD_compmean_). Data are given as means ± standard deviations. Configurations are unloaded (UL) and loaded to 147 N (LO), while conditions are sequential deficiencies of the lateral collateral ligament (LCL), popliteus tendon (PT), popliteofibular ligament (PFL), and anterior cruciate ligament (ACL). Statistical analysis of SCD values was based on repeated measures two-way ANOVA and significant differences are highlighted in bold type. p-values are reported for the comparison of all conditions and configurations. Select post-hoc details are provided in Supplementary Table 1.

	Intact		LCL deficiency		LCL and PT deficiency		LCL, PT, and PFL deficiency		LCL, PT, PFL, and ACL deficiency		p values
	UL	LO	UL	LO	UL	LO	UL	LO	UL	LO	
**Manual reference measures**											
SCD_manmean_	7.8 ± 1.4	9.9 ± 1.8	8.0 ± 1.5	11.2 ± 2.0	8.1 ± 1.3	11.7 ± 1.8	8.2 ± 1.4	12.1 ± 1.8	8.7 ± 1.2	12.5 ± 1.8	**< 0.01**
SCD_ml1_	6.6 ± 1.4	8.7 ± 1.6	6.6 ± 1.6	10.2 ± 1.8	7.0 ± 1.4	10.7 ± 1.8	6.9 ± 1.5	11.0 ± 1.7	7.4 ± 1.6	11.4 ± 1.8	
SCD_ml2_	6.6 ± 1.6	8.6 ± 1.7	6.7 ± 1.6	10.2 ± 2.0	6.8 ± 1.4	10.6 ± 1.9	7.1 ± 1.5	10.8 ± 1.9	7.5 ± 1.3	11.2 ± 2.0	
SCD_ml3_	7.0 ± 2.0	8.9 ± 2.4	7.2 ± 2.1	10.2 ± 2.6	7.3 ± 2.0	10.5 ± 2.4	7.5 ± 2.2	10.9 ± 2.4	8.1 ± 1.7	11.2 ± 2.4	
SCD_ap1_	10.5 ± 1.4	13.1 ± 2.1	10.7 ± 1.5	13.9 ± 2.4	10.6 ± 1.5	14.4 ± 2.4	11.1 ± 1.6	14.8 ± 2.4	11.7 ± 1.3	15.2 ± 2.2	**0.049**
SCD_ap2_	6.8 ± 1.6	9.0 ± 2.0	7.0 ± 1.7	10.3 ± 2.0	7.3 ± 1.4	10.7 ± 1.9	7.5 ± 1.5	11.2 ± 1.9	7.8 ± 1.4	11.4 ± 2.1	**< 0.01**
SCD_ap3_	9.0 ± 1.7	10.7 ± 1.8	9.5 ± 1.5	12.7 ± 1.6	9.5 ± 1.5	13.5 ± 1.5	9.4 ± 1.6	13.9 ± 1.7	9.5 ± 1.5	14.3 ± 1.9	
**Computational measures**											
SCD_compmean_	8.7 ± 1.4	10.8 ± 1.8	8.9 ± 1.6	12.4 ± 2.1	9.0 ± 1.3	12.7 ± 1.8	9.2 ± 1.2	13.0 ± 1.8	9.5 ± 1.3	13.5 ± 1.9	
SCD_ml1_	6.5 ± 1.2	8.7 ± 1.6	6.8 ± 1.5	10.4 ± 1.9	6.8 ± 1.4	10.8 ± 1.6	6.9 ± 1.3	11.1 ± 1.6	7.3 ± 1.5	11.6 ± 1.8	
SCD_ml2_	6.6 ± 1.4	8.7 ± 1.8	6.7 ± 1.6	10.0 ± 2.1	7.0 ± 1.4	10.4 ± 1.8	7.0 ± 1.3	10.9 ± 1.7	7.5 ± 1.3	11.3 ± 2.0	
SCD_ml3_	7.0 ± 2.2	9.0 ± 2.5	7.1 ± 2.2	10.0 ± 2.8	7.2 ± 1.9	10.5 ± 2.5	7.5 ± 1.9	10.6 ± 2.7	8.0 ± 1.8	11.1 ± 2.4	**0.01**
SCD_ap1_	10.8 ± 1.0	13.4 ± 2.2	10.9 ± 1.2	14.2 ± 2.2	10.9 ± 1.2	14.2 ± 1.9	11.1 ± 1.2	14.8 ± 2.0	11.8 ± 1.3	15.3 ± 2.2	0.254
SCD_ap2_	6.9 ± 1.2	9.0 ± 1.7	7.1 ± 1.4	10.4 ± 2.2	7.3 ± 1.4	10.5 ± 1.8	7.2 ± 1.2	11.1 ± 1.7	7.8 ± 1.2	11.6 ± 2.0	**< 0.01**
SCD_ap3_	7.4 ± 1.9	9.5 ± 1.7	7.9 ± 1.8	11.4 ± 2.1	7.8 ± 1.6	12.4 ± 1.8	8.2 ± 1.6	12.4 ± 2.0	8.0 ± 1.7	12.9 ± 1.9	

In contrast, absolute differences between the unloaded and loaded configurations increased with the increasing extent of injury. In the intact condition, averaged manual reference (SCD_manmean_) and averaged computational measures (SCD_compmean_) increased from 7.8 ± 1.4 mm and 8.7 ± 1.4 mm (unloaded) to 9.9 ± 1.8 mm and 10.8 ± 1.8 mm (loaded) (p < 0.001), respectively. After transection of the LCL, PT, and PFL, the respective values increased from 8.2 ± 1.4 mm and 9.2 ± 1.2 mm (unloaded) to 12.1 ± 1.8 mm and 13.0 ± 1.8 mm (loaded) (p < 0.001), while additional transection of the ACL led to further increases from 8.7 ± 1.2 mm and 9.5 ± 1.3 mm (unloaded) to 12.5 ± 1.8 mm and 13.5 ± 1.9 mm (loaded) (p < 0.001).

## Discussion

The most important findings of this study are that (i) MRI measures of lateral compartment opening under varus stress are related to the extent of injury of the PLC, thereby indicating a clear association between anatomic structure, injury, and functional consequences, and that (ii) lateral compartment opening may be similarly quantified using manual and computational methods alike.

In the clinical routine, MRI is well suited to assess acute injuries of the cruciate and collateral ligaments and to determine the extent and location of structural damage. Yet, MRI is oftentimes limited in assessing the complex and variable anatomy of the PLC. In particular, correct assessment of the static stabilizers such as the PFL, the fabellofibular and arcuate ligaments, and the popliteomeniscal fascicles remains challenging^25^. While the PFL is regularly present with reported prevalence rates of ≥ 94%, the fabellofibular and arcuate ligaments are notoriously variable and not regularly observed^25–27^, while the existence of the arcuate ligaments as distinct structures has been questioned repeatedly^26,28^. Consequently, the intraoperative assessment of the PLC is still considered the reference standard^29^, while consensus prevails that more functional techniques that apply varus stress must be developed to quantify lateral compartment opening reliably^30^.

Our study provides further evidence that lateral compartment opening provides a valuable surrogate marker for lateral knee (in)stability. When considering the unloaded configurations only, progressive injury of the PLC only marginally affected lateral compartment opening (in terms of SCDs), which is not of diagnostic use. Once varus stress was applied, changes in SCDs were significant. In LCL deficiency, the lateral compartment opened by an additional 1.3 mm (SCD_manmean_) and 1.6 mm (SCD_compmean_) as compared to the intact condition. This observation is well-aligned with parts of the literature as McDonald et al. reported similar differences of 1.48 mm or 1.99 mm under 12 Nm and clinician-applied loading, respectively, and combined LCL and ACL injuries of human cadaveric knees^16^. Other comparable studies, however, reported substantially higher differences. In a cadaveric study performed at 30° of flexion, LaPrade et al. quantified the amount of additional opening as 2.1 mm and 2.7 mm when subjecting the joint to 12 Nm and clinician-applied loading^15^. In a patient study using 20° of flexion and loading of 9 kg, Jacobsen et al. confirmed these findings and concluded that an increase in lateral compartment opening of > 2 mm versus the contralateral joint was diagnostic of an LCL injury^3^. This range was later confirmed again in other clinical studies, too^24,31^. In combined LCL, PT, and PFL deficiency, i.e., complete injury of the PLC, the lateral compartment opened by an additional 2.2 mm (SCD_manmean_ and SCD_compmean_) as compared to the intact condition which is, again, substantially lower than in other comparable studies. For example, LaPrade et al. reported mean increases of 3.4 mm and 4.0 mm (12 Nm and clinician-applied loading)^15^, while Crawford et al. reported a mean increase of 4.9 mm (cadaveric study, 30° of flexion, 12 Nm, arthroscopic measurements)^31^. After additional transection of the ACL, i.e., in combined PLC and ACL deficiency, the lateral compartment opened by an additional 2.6 mm (SCD_manmean_) and 2.7 mm (SCD_compmean_) as compared to the intact condition, which is, again, well in line with the smaller differences of 1.9 mm and 2.7 mm (12 Nm and clinician-applied loading) reported by McDonald et al.^16^, yet substantially lower than the differences reported by LaPrade et al.^15^ (i.e., 5.3 mm and 6.6 mm) or by Crawford et al.^31^ (i.e., 8.8 mm).

Taken together, current literature findings unambiguously indicate that lateral compartment opening increases with the increasing extent of PLC injury. Yet, despite largely similar (or even largely identical) experimental setups, variability in absolute values is substantial and likely due to a variety of contributing factors. These include variable imaging modalities (i.e., radiography vs. arthroscopy vs. MRI) and measurement procedures (i.e., single-location vs. averaged multi-location measurements, exact measurement locations), as well as differences in joint position (i.e., 20° vs. 30° vs. > 30° of flexion), load application (i.e., physician-applied vs. instrumented loading), and load magnitude (i.e., 12 Nm vs. 9 kg vs. 15 kp).

Radiographs are particularly prone to inaccuracy even though they are widely considered the reference standard modality for imaging under varus stress^17^. Differences in joint position have a large impact on the radiographic appearance of the joint space because (i) 2D projections are taken of 3D structures^32^, (ii) even slightly different X-ray beam angles produce substantial differences in joint spaces^33^, and (iii) changes in cartilage and meniscus affect the joint space, too, yet are unaccounted for^32^. In contrast, MRI as the state-of-the-art imaging modality of contemporary musculoskeletal imaging is well-suited to delineate the lateral compartment’s joint space in 3D and as a function of femorotibial flexion, rotation, and translation. In our study, spatial assessment of joint space opening indicated that loading-induced increases were largest posteriorly. This is in line with the literature as the PLC also resists posterolateral rotation of the tibia relative to the femur^26,28^. Consequently, relatively increased opening of the posterior lateral compartment is a sign of the joint’s compromised rotatory stability and provides a promising diagnostic target for future research efforts.

We also investigated the potential diagnostic value of computational versus manual reference measurements. When determined at corresponding single locations, SCD values were largely similar and differences only marginal which may be due to methodological differences. Manual reference measurements were performed on the 3D proton density-weighted fat-suppressed sequences, while the T1-weighted sequences were used for segmentation and computation of the 3D joint models. In this regard, the exact delineation of the cartilage-bone transition and the accurate subsequent computation of the SCDs may be challenged by the presence of chemical shift artifacts, especially at the posterior femoral condyles, that depend on the receiver bandwidth and phase-encoding direction. Even though alternative MRI approaches could quantify lateral joint opening based on cartilage surfaces, we -intentionally- included the cartilage tissue (in the concept of SCD computation) to allow for comparisons with radiographic measures. When averaged across the entire lateral joint compartment, computationally determined SCD values were larger than manually determined SCD values which is likely because larger areas of the joint were included. Also, averaged computational measurements are beneficial because they (i) are less prone to incorrect measurements, thereby improving accuracy, (ii) average over large joint areas, thereby improving robustness to outliers, and (iii) relieve the radiologist and/or orthopaedist from tedious measurement tasks. Yet, to date, the developed 3D joint models rely on accurate segmentations which are too laborious for clinical practice and require automation in the future^34^.

For the future clinical application, several aspects must be considered once regulatory and medicolegal challenges are overcome. Device operability and safety, positioning and fixation of the coils, joint, and actuator, loading-induced motion, patient comfort, and measurement validity and reproducibility need to be studied with the device in clinical operation. With regards to oftentimes tight MRI schedules, examination times need to be as short as possible to balance the additional diagnostic value against the additional time demand. Our study was set up to be as reflective of the actual in-vivo situation as possible—by using clinical sequence parameters, coils, hardware, and a 3 T scanner, we hope to boost the future clinical translation of stress MRI techniques in functional joint assessment.

This study has several limitations. First, it was conducted ex vivo using human cadaveric specimens. Thus, we only evaluated the joint’s passive stabilizers, while dynamic stabilizers such as muscles could not be assessed^1,35^. Second, the ligamentous injuries were created by surgical transection which fails to emulate the complex mechanisms of injury in vivo that also involve additional traumatic injuries to bone, soft tissues, and/or other stabilizers. Consequently, clinically relevant concomitant injuries of the iliotibial band, biceps femoris, lateral gastrocnemius, anterolateral ligament, fabellofibular ligament, and lateral meniscus^1,6,7,14^ were not considered in our injury model. Third, the effects of standardized injury were compared to the intact condition as is common in this type of research^15,31^, while, clinically, the side-to-side differences versus the non-injured joint are assessed. Fourth, the lateral joint space was not referenced to instrumented laxity measurements or clinical tests that are commonly applied in the clinic. Fifth, the normative values we established in body donors of advanced age may be of limited relevance to the younger clinical patient cohort because of potentially altered tissue stiffness.

In conclusion, varus stress MRI brings together advanced imaging and functional evaluation and allows comprehensive assessment of the PLC—by direct assessment of its structural integrity and by indirect assessment of its functional capacity. Once set up, sophisticated image post-processing techniques are equally reliable as manual reference measurements yet require further refinement to streamline the segmentation process for clinical practice. Beyond, this study provides normative values of lateral compartment opening as a function of PLC injury and may thus be useful for enhanced diagnosis of acute and chronic lateral joint instability as well as therapeutic decision-making and treatment monitoring in the future.

## Materials and methods

### Study design and sample size estimation

This study was designed as an experimental in-situ imaging study on human cadaveric knee joint specimens. Based on local institutional Review Board approval (Ethical Committee, RWTH Aachen University, EK180/16, issued 07/13/2016), this study was carried out between 04/2021 and 07/2021. All methods were carried out in accordance with relevant guidelines and regulations. Written informed consent by the body donors and next of kin or their legal guardians was obtained. Minimum specimen size of 10 was determined based on power analyses of the initial three specimens and dedicated online software (www.statstodo.com): power, 0.8; probability of type-I-error, 0.01; effect size, 1.4, two-tailed procedure.

In total, ten unpaired, left-sided, fresh-frozen human cadaveric knee joint specimens were obtained from the local Anatomy department (Institute of Molecular and Cellular Anatomy, RWTH Aachen University). The body donors were five males and five females with a mean age of 80 ± 10 years (range 65–95 years). Before measurements, specimens were left to thaw at room temperature for at least 24 h.

### Stress MRI device for pressure controlled varus loading

**Figure 3:**
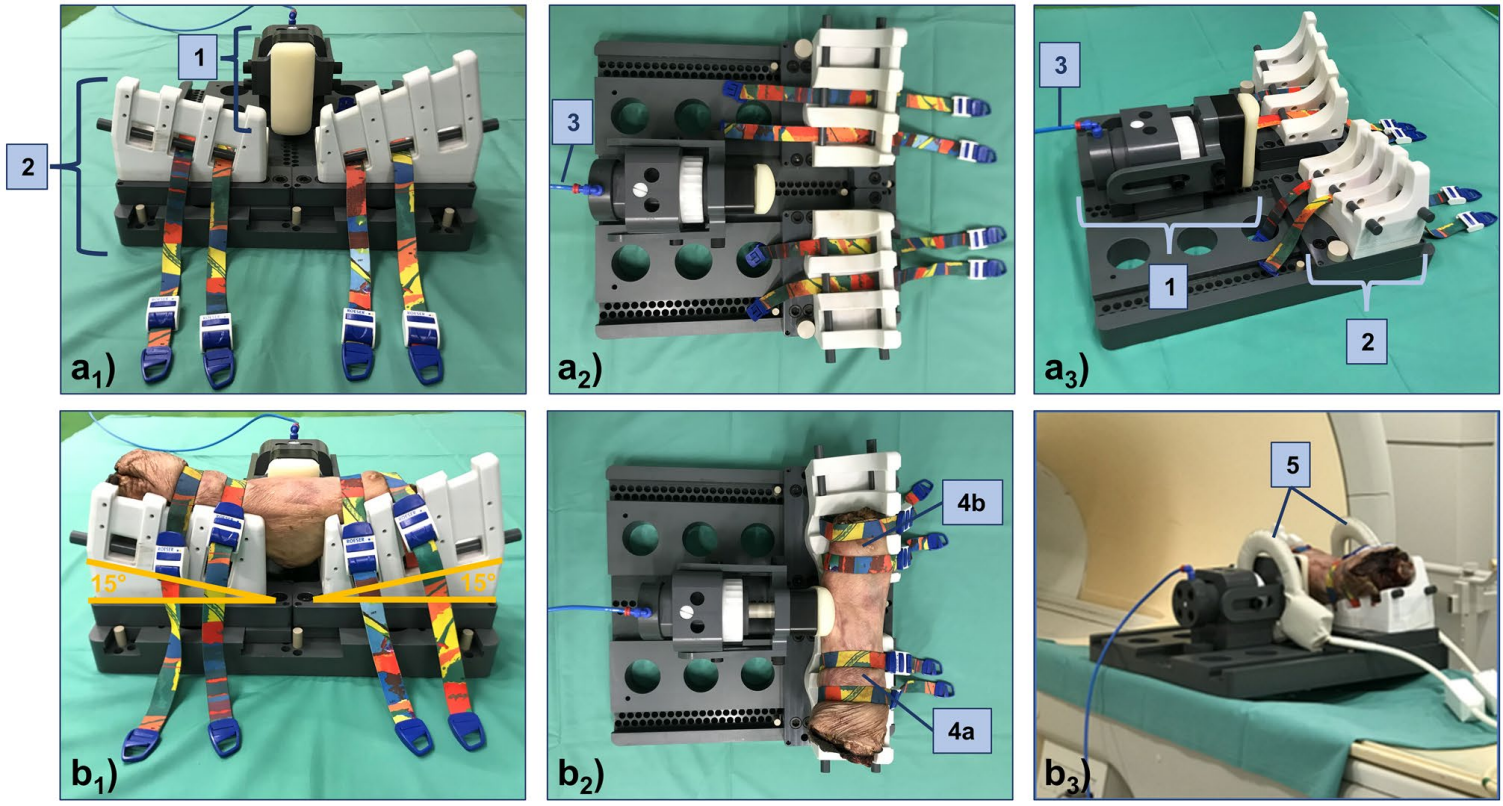
Setup for varus stress MRI. The MRI-compatible loading device is shown without (**a**) and with (**b**) a representative (left-sided) knee joint specimen. Side views (**a**_**1**_, **b**_**1**_), top views (**a**_**2**_, **b**_**2**_), and oblique views (**a**_**3**_, **b**_**3**_). Loading unit (1), holding unit (2), standard pressure line (3) to connect the loading unit and the control unit (latter one not shown). Upper (4a) and lower (4b) thigh at 30° of flexion (patella facing downwards). Dual coils (5) were used for imaging.

To standardize joint position and load application, a pressure-controlled MRI-compatible loading device as validated before^36,37^ was used. The device has three functional units, i.e., the holding unit, the loading unit, and the control unit (Fig. 3). Briefly, the holding unit consists of two equally sized quarter-pipe wedges of 15° inclination each that standardize joint flexion at 30°. The specimens were positioned on the wedges with the patella facing downwards, corresponding to a patient’s prone position, and fixed using four standard tourniquets. The loading unit consists of a padded load applicator that is actuated through a pneumatic mechanism and controlled by pressure. Practically, the load applicator was aligned along the specimens’ medial joint line so that, once pressure was applied, the medial compartment was compressed, and the lateral compartment was distracted. The control unit was located outside of the scanner room and connected via standard pressure lines. Customized routines implemented in LabView software (v2020, National Instruments, Austin, US) were used to control pressure levels.

### Graded injury model of the posterolateral corner and the anterior cruciate ligament

**Figure 4:**
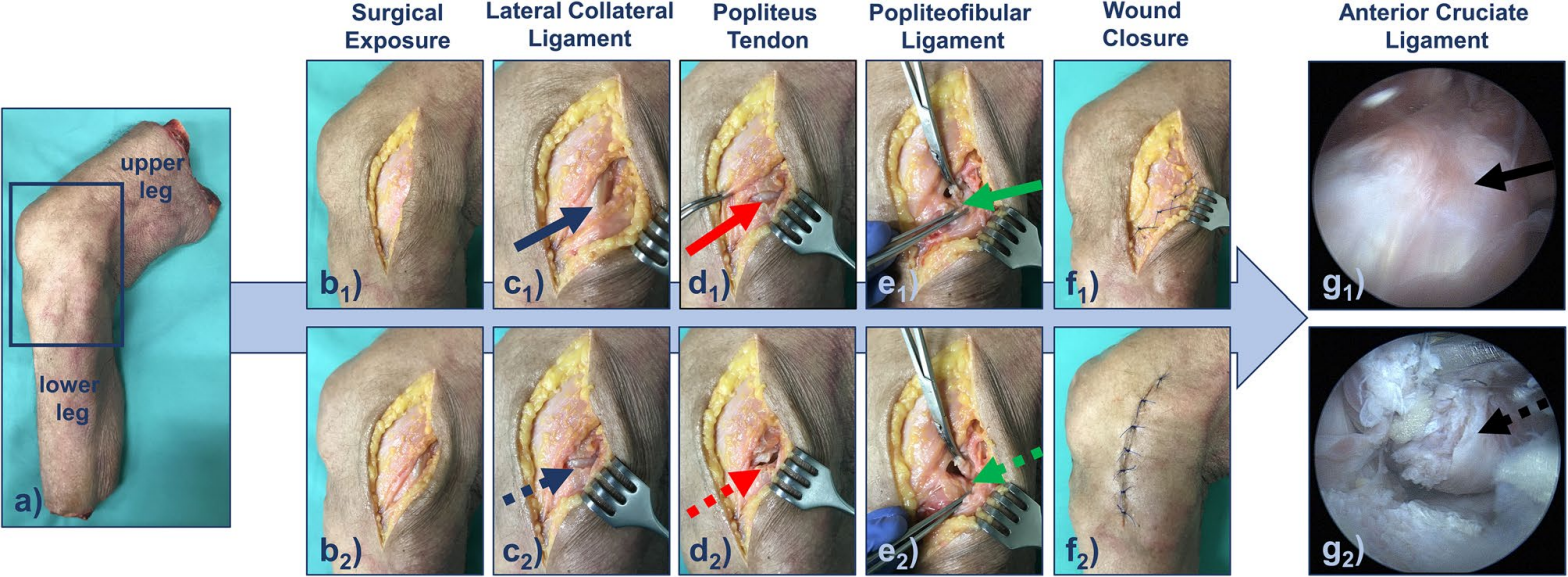
Graded injury model of the posterolateral corner and the anterior cruciate ligament. The joint (**a**) was subjected to standardized sequential surgical transections. After exposure (**b**_**1**_) and splitting of the fascia (**b**_**2**_), the lateral collateral ligament (blue arrow, **c**_**1**_), the popliteal tendon (red arrow, **d**_**1**_), and the popliteofibular ligament (green arrow, **e**_**1**_) were exposed. Sequentially, these structures were surgically transected to emulate complete LCL injury (dotted blue arrow, **c**_**2**_), additional complete PT injury (dotted red arrow, **d**_**2**_), and additional complete PFL injury (green dotted arrow, **e**_**2**_). Each transection was followed by layer-wise adaptation and suturing of the fascia (**f**_**1**_) and skin (**f**_**2**_). Arthroscopically, the anterior cruciate ligament was identified (black arrow, **g**_**1**_) and completely transected at mid-substance (dotted black arrow, **g**_**2**_).

The injury model was focused on the three main structures of the PLC, i.e., the LCL, the PT, and the PFL. In addition, the injury model also involved the ACL because of its clinical relevance as a concomitant injury in PLC injuries^8^. Figure 4 details the sequential ligament transections.

The LCL, the PT, and the PFL were sequentially transected and this order was chosen based on previously published studies^15,31^. The surgical approach to expose the PLC involved posterolateral splitting of the fascia as described by Terry and LaPrade^38^. Briefly, the knee joint was exposed using a slightly curved skin incision between the fibular head and Gerdy’s tubercle (Fig. 4b1). Once the skin and fat were retracted, the fascia was split parallel to the iliotibial tract (Fig. 4b2) to expose the LCL (Fig. 4c1). The LCL was transected at the level of the joint space (i.e., at mid-substance) (Fig. 4c2). Subsequently, the PT (Fig. 4d1) and the PFL (Fig. 4e1) were identified and sequentially transected, too, i.e., the PT first (Fig. 4d2) and the PFL second (Fig. 4e2). After each individual transection, the fascia and skin were adapted and sutured in a layer-wise manner (Fig. 4f). Of note, the status of completely transected LCL, PT, and PFL is referred to as complete PLC injury.

The ACL was transected during standard arthroscopy. Following placement of the anteromedial and anterolateral portals, the ACL was identified (Fig. 4g1) and transected at its mid-substance (Fig. 4g2) using arthroscopic straight-tip scissors (Arthrex, Naples, FL, US). After thorough irrigation and clearing of excess fluid, the portals were sutured.

Consequently, each knee joint specimen was evaluated in five conditions:

(i) intact;
(ii) deficiency of the LCL;
(iii) deficiency of the LCL and PT;
(iv) deficiency of the LCL, PT, and PFL;
(v) deficiency of the LCL, PT, PFL, and ACL.

**Figure 5:**
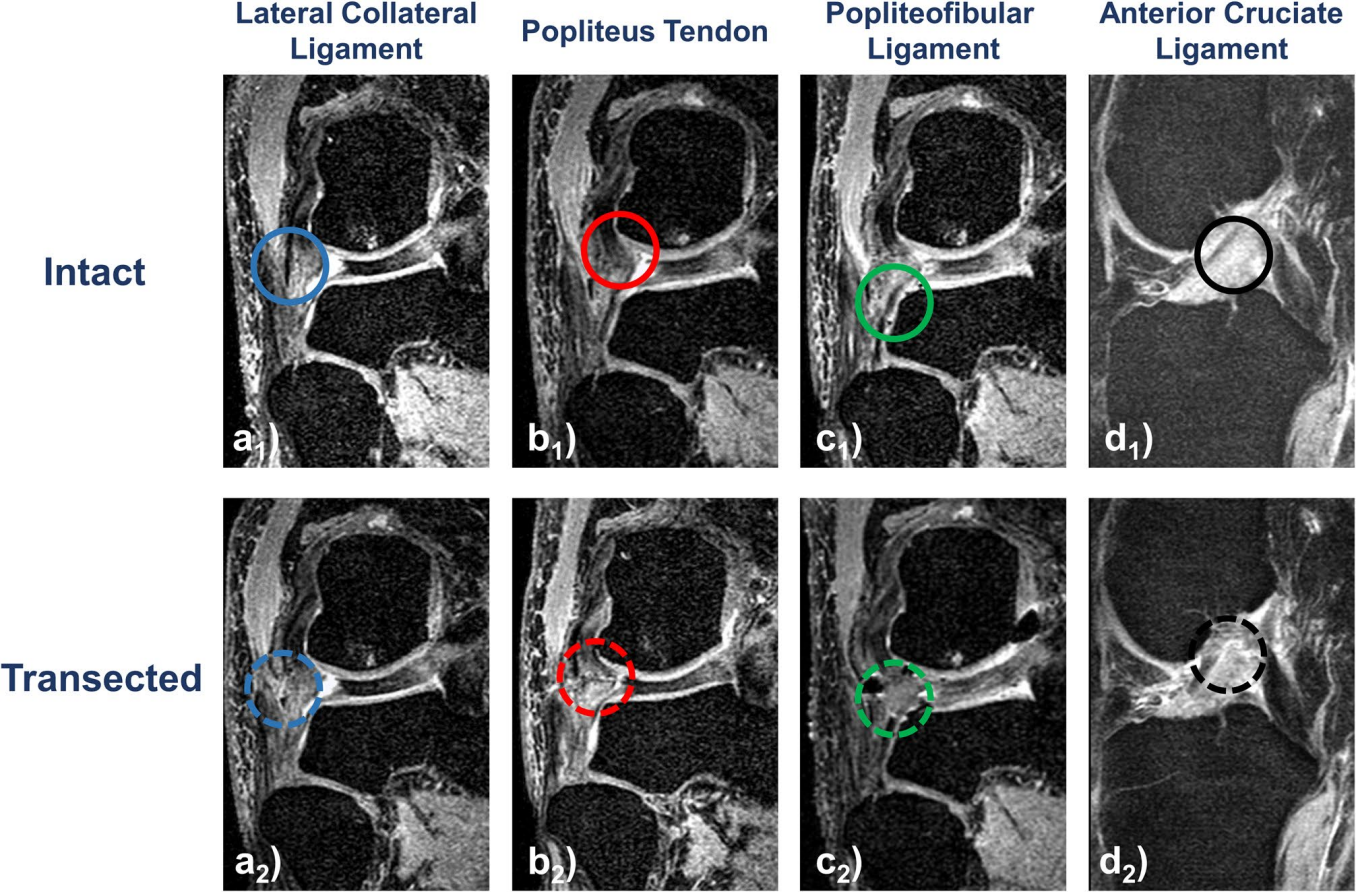
Morphologic MR images of the graded injury model. Visualized are representative coronal (**a**–**c**) and sagittal (**d**) images centred on the respective structure before (“intact” [**a**_**1**_–**d**_**1**_], full circles) and after sequential surgical transection (“transected” [**a**_**2**_–**d**_**2**_], dotted circles). 3D proton density-weighted fat-saturated sequence. Color-coding: lateral collateral ligament—blue, popliteus tendon—red, popliteofibular ligament—green.

Figure 5 visualizes the graded injury model. The respective anatomic structure’s integrity (in the intact condition) and transected states (in the deficient conditions) were directly assessed by a clinical radiologist (S.N., 8 years of experience).

### MR image acquisition

MR imaging was performed at room temperature using a clinical 3.0 T scanner (Achieva, Philips, Best, The Netherlands). After loading the device with the specimen, flexible multi-purpose phased-array receive-only dual-coils (Sense Flex-M, Philips) were positioned at the medial and lateral aspects of the joint (Fig. 3b3).

The imaging protocol consisted of scout views, 2D T1-, and 2D T2-weighted turbo spin-echo sequences, and a 3D proton density-weighted sequence with fat suppression (Supplementary Table 2). The imaging protocol was completed for each specimen, configuration (i.e., unloaded and loaded), and condition (i.e., five conditions ranging from intact to completely PLC and ACL deficient). Consequently, the imaging protocol was acquired ten times per specimen. Magnet time per specimen, configuration, and condition was approximately 20 min. The total magnet time per specimen was approximately 3.5 h.

**Figure 6:**
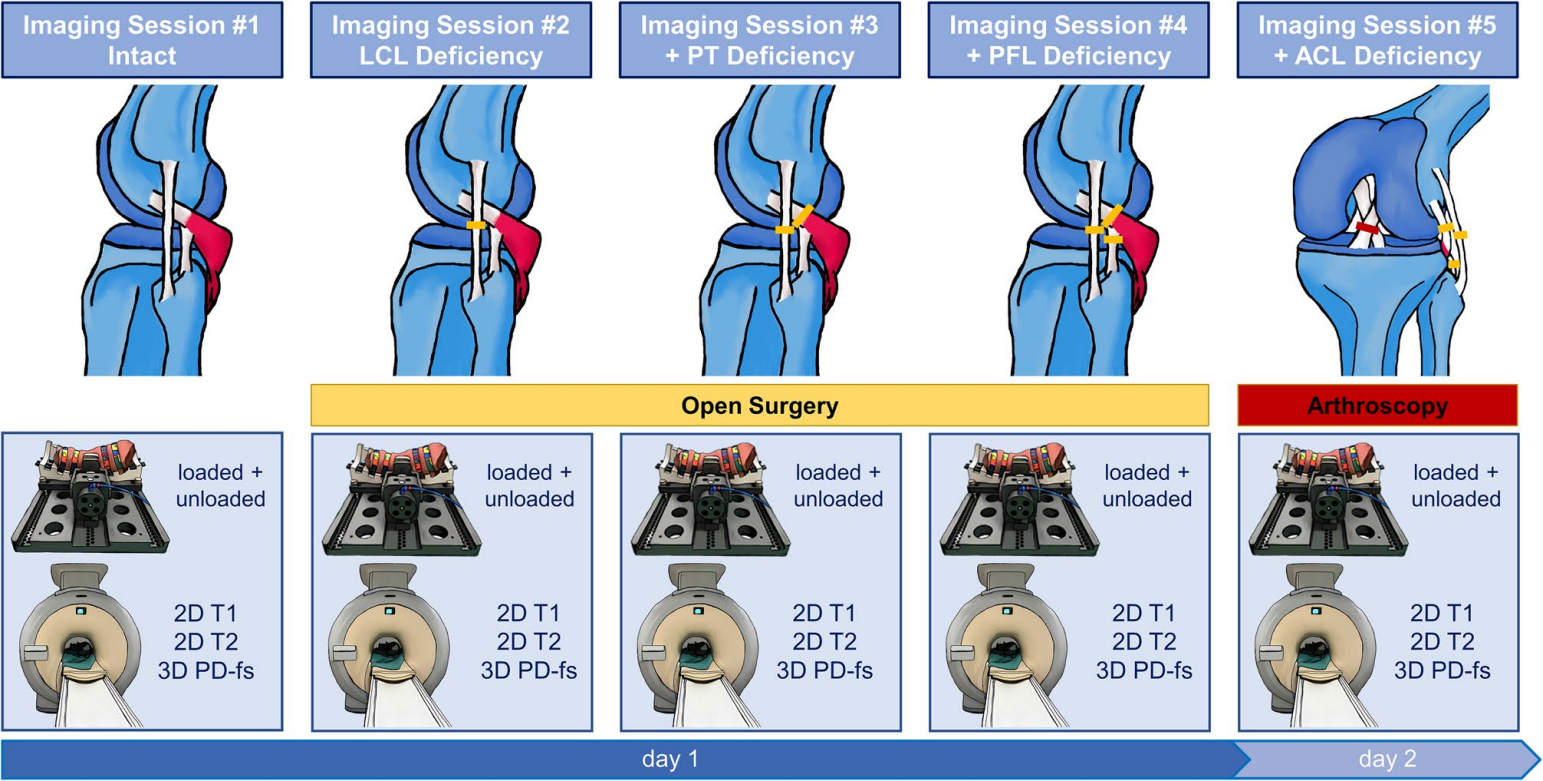
Timeline of imaging sessions as a function of graded injury. The lateral collateral ligament (LCL), the popliteal tendon (PT), the popliteofibular ligament (PFL), and the anterior cruciate ligament (ACL) were transected via open surgical (orange) and arthroscopic approaches (red), respectively. T1-weighted, T2-weighted, and proton density-weighted fat-saturated sequences were used to image the joints in each condition and configuration.

The timeline of the imaging sessions is detailed in Fig. 6. Particular attention was paid to intra- and inter-individually standardize loading and imaging conditions for each imaging session.

### Manual reference measurements

Two readers (M.C. and E.-M. W., medical students in their final year with 2 years of experience in musculoskeletal imaging each) performed the manual 2D reference measurements of lateral compartment opening. Since loading configurations and joint conditions were readily distinguishable, the readers were not blinded to each specimen’s status. Using the in-house picture archiving and communication system (iSite, Philips Healthcare, Amsterdam, The Netherlands), its standard image analysis toolbox, and the 3D proton density-weighted sequence with fat suppression, the vertical distances between the femoral und tibial subchondral cortices were quantified as SCDs (subchondral cortical distances). SCDs were determined in the anteroposterior and mediolateral dimensions at three locations each (Supplementary Fig. 1). Referred to as SCD_ml1_ to SCD_ml3_ (from lateral to medial), mediolateral SCD measurements were performed on the mid-coronal images after dividing the lateral tibial condyle into quarters to define the lateral (SCD_ml1_), central (SCD_ml2_), and medial (SCD_ml3_) measurement locations. Referred to as SCD_ap1_ to SCD_ap3_ (from anterior to posterior), anteroposterior SCD measurements were performed on the mid-sagittal image of the lateral compartment at the base of the anterior horn of the lateral meniscus (SCD_ap1_), at the center of the lateral meniscus body (SCD_ap2_), and at the base of the posterior horn of the lateral meniscus (SCD_ap3_). SCD measures were determined for each specimen, configuration, and condition.

Inter-reader agreement was determined using the intraclass correlation coefficient (online calculator v1.5, Mangold International, Arnstorf, Germany). Because inter-reader agreement was excellent with intraclass correlation coefficients of ≥ 0.95, i.e., 0.98 (SCD_ml1_), 0.99 (SCD_ml2_), 0.97 (SCD_ml3_), 0.96 (SCD_ap1_), 0.99 (SCD_ap2_), and 0.96 (SCD_ap3_) (single scorings, not adjusted), the manual reference measurements of both readers were pooled and averaged SCD_manmean_.

### MR image post-processing and computational measurements

Computational measurements were based on manual segmentations as before^36^. Briefly, for each specimen, configuration, and condition, the femoral and tibial bone outlines were manually segmented on coronal T1-weighted images using the semiautomatic segmentation function of ITK-SNAP (v3.8, Cognitica, Philadelphia, PA, US)^39^. The medial and lateral tibial condyles were labeled separately to provide spatially defined measurements of lateral compartment opening (Supplementary Fig. 2).

**Figure 7:**
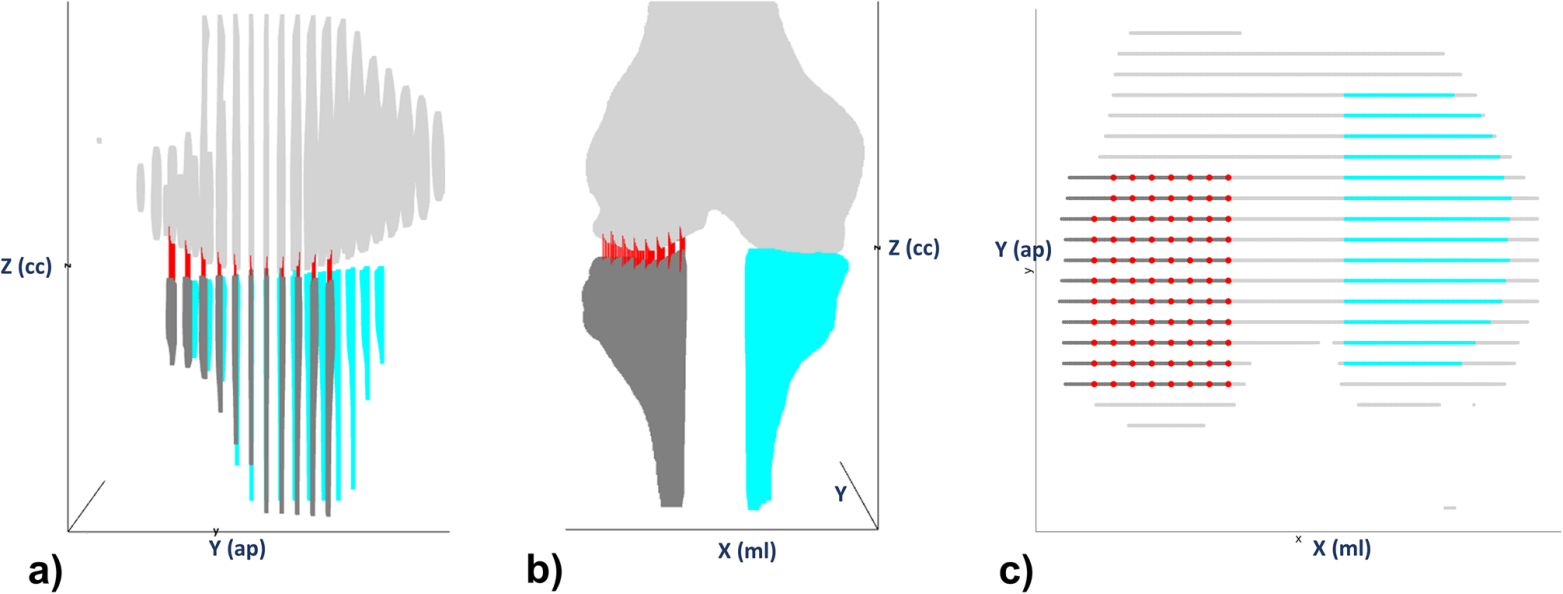
Computational measurements of subchondral cortical distances based on joint-specific 3D models. After manual segmentations of the femur (light grey) and the tibia (dark grey [lateral]; turquoise [medial]), the segmentation outlines were transferred to specific Cartesian coordinate systems (angulated along the z-axis as defined by the tibial bone axis) and used as 3D models for each joint, configuration, and condition. Grids with fixed spacings were used to standardize measurement locations of the subchondral cortical distances (red lines) that were computed parallel to the z-axis. X-, y-, and z-axes indicate the mediolateral (ml), anteroposterior (ap), and craniocaudal (cc) dimensions. Accordingly, the y–z-, x–z-, and x–y-planes represent the sagittal (**a**), coronal (**b**), and axial orientations (**c**). The sliced appearance of the 3D models in (**a**) and (**c**) is due to interslice gaps.

Segmentation outlines were transferred into specific Cartesian coordinate systems where the x-axis, y-axis, and z-axis represent the mediolateral, anteroposterior, and craniocaudal dimensions. These outlines were used as 3D representations of the joints in each configuration and condition (Fig. 7). The surfaces of the distal femur and the proximal tibia were delineated by pre-processing and used for subsequent SCD measurements. Then, customized grids with regular spacings of 3.3 mm (anteroposterior) and 3.5 mm (mediolateral) were defined for the lateral tibial condyle and used to align the SCD measurements along the grid points and parallel to the z-axis. Consequently, the vertical distances between the femoral and tibial coordinate hulls were determined. Of note, the lateral grid points (corresponding to the lateralmost 10% of the femur’s maximum diameter along the transepicondylar axis) were automatically excluded to prevent erroneous SCD measurements due to the shape of the femoral epicondyle. In total, 73 ± 12 (mean ± standard deviation) SCD measurements along the grid points were obtained per joint, configuration, and condition and averaged as SCD_compmean_.

For intra-individual comparisons, the grids of all subsequent configurations and conditions were referenced to the baseline condition and configuration, i.e., intact and unloaded.

To allow for comparisons with the manual reference measurements (SCD_ml1_ to SCD_ml3_ and SCD_ap1_ to SCD_ap3_), corresponding computational SCD measurement locations were identified.

### Statistical analysis

Statistical analysis was performed by M.C. and S.N. using GraphPadPrism (v9.1, San Diego, CA, US). Assuming underlying normal distributions, repeated measures two-way ANOVA was used to compare mean SCD values across the different configurations and conditions. Pairwise posthoc comparisons were performed for the mean SCD values using Tukey’s test. Throughout, multiplicity-adjusted p-values are indicated to account for multiple comparisons against the family-wise alpha error threshold of p ≤ 0.05.

## Supplementary Information

Supplementary Information.

## Data Availability

The datasets generated and analyzed in this study are available from the corresponding author on reasonable request.

## References

[1] James EW, LaPrade CM, LaPrade RF (2015). Anatomy and biomechanics of the lateral side of the knee and surgical implications. Sports Med. Arthrosc. Rev..

[2] Klein KK (1962). An instrument for testing the medial and lateral collateral ligament stability of the knee. Am. J. Surg..

[3] Jacobsen K (1976). Stress radiographical measurement of the anteroposterior, medial and lateral stability of the knee joint. Acta Orthop. Scand..

[4] Gollehon DL, Torzilli PA, Warren RF (1987). The role of the posterolateral and cruciate ligaments in the stability of the human knee. A biomechanical study. J. Bone Jt. Surg. Am..

[5] Grood ES, Stowers SF, Noyes FR (1988). Limits of movement in the human knee. Effect of sectioning the posterior cruciate ligament and posterolateral structures. J. Bone Jt. Surg. Am..

[6] LaPrade RF, Wentorf FA, Fritts H, Gundry C, Hightower CD (2007). A prospective magnetic resonance imaging study of the incidence of posterolateral and multiple ligament injuries in acute knee injuries presenting with a hemarthrosis. Arthrosc. J. Arthrosc. Relat. Surg..

[7] Temponi EF, de Carvalho LH, Saithna A, Thaunat M, Sonnery-Cottet B (2017). Incidence and MRI characterization of the spectrum of posterolateral corner injuries occurring in association with ACL rupture. Skelet. Radiol..

[8] Becker EH, Watson JD, Dreese JC (2013). Investigation of multiligamentous knee injury patterns with associated injuries presenting at a level I trauma center. J. Orthop. Trauma.

[9] Vinson EN, Major NM, Helms CA (2008). The posterolateral corner of the knee. AJR Am. J. Roentgenol..

[10] Devitt BM, Whelan DB (2015). Physical examination and imaging of the lateral collateral ligament and posterolateral corner of the knee. Sports Med. Arthrosc. Rev..

[11] Pacheco RJ, Ayre CA, Bollen SR (2011). Posterolateral corner injuries of the knee: A serious injury commonly missed. J. Bone Jt. Surg. Br..

[12] Chahla J (2019). Posterolateral corner of the knee: An expert consensus statement on diagnosis, classification, treatment, and rehabilitation. Knee Surg. Sports Traumatol. Arthrosc..

[13] Collins MS (2015). MRI injury patterns in surgically confirmed and reconstructed posterolateral corner knee injuries. Knee Surg. Sports Traumatol. Arthrosc..

[14] Geeslin AG, LaPrade RF (2010). Location of bone bruises and other osseous injuries associated with acute grade III isolated and combined posterolateral knee injuries. Am. J. Sports Med..

[15] LaPrade RF, Heikes C, Bakker AJ, Jakobsen RB (2008). The reproducibility and repeatability of varus stress radiographs in the assessment of isolated fibular collateral ligament and grade-III posterolateral knee injuries. An in vitro biomechanical study. J. Bone Jt. Surg. Am..

[16] McDonald LS (2016). Validation of varus stress radiographs for anterior cruciate ligament and posterolateral corner knee injuries: A biomechanical study. Knee.

[17] James EW, Williams BT, LaPrade RF (2014). Stress radiography for the diagnosis of knee ligament injuries: A systematic review. Clin. Orthop. Relat. Res..

[18] Ross G, Chapman AW, Newberg AR, Scheller AD (1997). Magnetic resonance imaging for the evaluation of acute posterolateral complex injuries of the knee. Am. J. Sports Med..

[19] Naraghi AM, White LM (2016). Imaging of athletic injuries of knee ligaments and menisci: Sports imaging series. Radiology.

[20] LaPrade RF, Gilbert TJ, Bollom TS, Wentorf F, Chaljub G (2000). The magnetic resonance imaging appearance of individual structures of the posterolateral knee. A prospective study of normal knees and knees with surgically verified grade III injuries. Am. J. Sports Med..

[21] Harish S, O'Donnell P, Connell D, Saifuddin A (2006). Imaging of the posterolateral corner of the knee. Clin. Radiol..

[22] Chien A, Weaver JS, Kinne E, Omar I (2020). Magnetic resonance imaging of the knee. Pol. J. Radiol..

[23] Bolog N, Hodler J (2007). MR imaging of the posterolateral corner of the knee. Skelet. Radiol..

[24] Kane PW (2018). Increased accuracy of varus stress radiographs versus magnetic resonance imaging in diagnosing fibular collateral ligament grade III tears. Arthrosc. J. Arthrosc. Relat. Surg..

[25] Ahn SJ (2016). Using three-dimensional isotropic SPACE MRI to detect posterolateral corner injury of the knee. Acta Radiol..

[26] Geiger D, Chang E, Pathria M, Chung CB (2013). Posterolateral and posteromedial corner injuries of the knee. Radiol. Clin..

[27] Munshi M (2003). MR imaging, MR arthrography, and specimen correlation of the posterolateral corner of the knee: An anatomic study. Am. J. Roentgenol..

[28] Dean RS, LaPrade RF (2020). ACL and posterolateral corner injuries. Curr. Rev. Musculoskelet. Med..

[29] Bonadio MB (2014). Correlation between magnetic resonance imaging and physical exam in assessment of injuries to posterolateral corner of the knee. Acta Ortopedica Brasileira.

[30] LaPrade RF, Heikes C, Bakker AJ, Jakobsen RB (2008). The reproducibility and repeatability of varus stress radiographs in the assessment of isolated fibular collateral ligament and grade-III posterolateral knee injuries: An in vitro biomechanical study. JBJS.

[31] Crawford B, Zehnder S, Cutuk A, Farrow LD, Kaar SG (2013). Arthroscopic evaluation of knee lateral compartment widening after lateral ligamentous injury. Knee Surg. Sports Traumatol. Arthrosc..

[32] Segal NA (2017). Comparison of tibiofemoral joint space width measurements from standing CT and fixed flexion radiography. J. Orthop. Res..

[33] Charles, H., Kraus, V., Ainslie, M. & Le Graverand-Gastineau, M.-P. H. Vol. 15 1221–1224 (Elsevier, 2007). 10.1016/j.joca.2007.05.012 17977754

[34] Schock, J. *et al.* In *International Workshop on Shape in Medical Imaging.* 85–94 (Springer).

[35] Rosas HG (2016). Unraveling the posterolateral corner of the knee. Radiogr. Rev. Publ. Radiol. Soc. N. Am..

[36] Ciba M (2021). Comprehensive assessment of medial knee joint instability by valgus stress MRI. Diagnostics (Basel)..

[37] Said O (2020). An MRI-compatible varus-valgus loading device for whole-knee joint functionality assessment based on compartmental compression: A proof-of-concept study. Magma (New York, NY).

[38] Terry GC, LaPrade RF (1996). The posterolateral aspect of the knee. Anatomy and surgical approach. Am. J. Sports Med..

[39] Yushkevich PA (2006). User-guided 3D active contour segmentation of anatomical structures: Significantly improved efficiency and reliability. Neuroimage.

