# Supplementary material for: Varus stress MRI in the refined assessment of the posterolateral corner of the knee joint
Source: Sci Rep. 2022 Jul 13;12:11858. doi: 10.1038/s41598-022-15787-2 (PMC9279472; doi:10.1038/s41598-022-15787-2)
Supplement: Supplementary file 1 — Supplementary Information. [file 41598_2022_15787_MOESM1_ESM.pdf]

**Supplementary Material**

**Title:** Varus Stress MRI in the Refined Assessment of the Posterolateral Corner of the Knee Joint

**Authors:** Malin Ciba, Eva-Maria Winkelmeyer, Justus Schock, Simon Westfechtel, Teresa Nolte, Matthias Knobe, Andreas Prescher, Christiane Kuhl, Daniel Truhn, Sven Nebelung

**Journal:** Scientific Reports

## Supplementary Tables

**Supplementary Table 1:** Pairwise post-hoc comparisons of manual reference and computational measurements of subchondral cortical distances (SCDs). Following repeated measures two-way ANOVA, Tukey's multiple comparisons test was performed to assess significant differences in SCDs as a function of loading configuration and joint condition. The family-wise alpha threshold was set to $p<.05$. Significant results are indicated in **bold type**. $SCD_{compmean}$ values refer to the mean SCD values (per joint, configuration, and condition) as determined computationally (i.e., based on joint-specific 3D models and averaged across the entire lateral joint compartment), while $SCD_{manmean}$ values refer to the mean SCD values (per joint, configuration, and condition) as determined by manual reference measurements. Abbreviations are detailed in **Table 1**.

| **Pairwise post-hoc comparison** | **Manual Reference Measures $SCD_{manmean}$** | **Computational Measures $SCD_{compmean}$** |
|---|---|---|
| **Intact UL vs. Intact LO** | **<0.001** | **<0.001** |
| **Intact UL vs. LCL-deficient UL** | 0.341 | 0.473 |
| **Intact UL vs. LCL-/PT-deficient UL** | 0.067 | 0.097 |
| **Intact UL vs. LCL-/PT-/PFL-deficient UL** | **0.041** | 0.059 |
| **Intact UL vs. LCL-/PT-/PFL-/ACL-deficient UL** | **0.003** | **0.003** |
| **Intact LO vs. LCL-deficient LO** | **<0.001** | **<0.001** |
| **Intact LO vs. LCL-/PT-deficient LO** | **<0.001** | **<0.001** |
| **Intact LO vs. LCL-/PT-/PFL-deficient LO** | **<0.001** | **<0.001** |
| **Intact LO vs. LCL-/PT-/PFL-/ACL-deficient LO** | **<0.001** | **<0.001** |
| **LCL-deficient UL vs. LCL-deficient LO** | **<0.001** | **<0.001** |
| **LCL-deficient UL vs. LCL-/PT-deficient UL** | 0.988 | 0.999 |
| **LCL-deficient UL vs. LCL-/PT-/PFL-deficient UL** | 0.558 | 0.824 |
| **LCL-deficient UL vs. LCL-/PT-/PFL-/ACL-deficient UL** | **0.029** | 0.153 |
| **LCL-deficient LO vs. LCL-/PT-deficient LO** | **0.012** | 0.243 |
| **LCL-deficient LO vs. LCL-/PT-/PFL-deficient LO** | **0.011** | 0.060 |
| **LCL-deficient LO vs. LCL-/PT-/PFL-/ACL-deficient LO** | **0.003** | **0.007** |
| **LCL-/PT-deficient UL vs. LCL-/PT-deficient LO** | **<0.001** | **<0.001** |
| **LCL-/PT-deficient UL vs. LCL-/PT-/PFL-deficient UL** | 0.959 | 0.745 |
| **LCL-/PT-deficient UL vs. LCL-/PT-/PFL-/ACL-deficient UL** | **0.046** | **0.018** |
| **LCL-/PT-deficient LO vs. LCL-/PT-/PFL-deficient LO** | 0.192 | 0.253 |
| **LCL-/PT-deficient LO vs. LCL-/PT-/PFL-/ACL-deficient LO** | **0.045** | 0.071 |
| **LCL-/PT-/PFL-deficient UL vs. LCL-/PT-/PFL-deficient LO** | **<0.001** | **<0.001** |
| **LCL-/PT-/PFL-deficient UL vs. LCL-/PT-/PFL-/ACL-deficient UL** | 0.464 | 0.621 |
| **LCL-/PT-/PFL-deficient LO vs. LCL-/PT-/PFL-/ACL-deficient LO** | 0.504 | 0.352 |
| **LCL-/PT-/PFL-/ACL-deficient UL vs. LCL-/PT-/PFL-/ACL-deficient LO** | **<0.001** | **<0.001** |

**Supplementary Table 2:** MRI Sequence Acquisition Parameters. Abbreviations: T1w – T1-weighted, T2w – T2-weighted, PDw fs – Proton Density-weighted with fat saturation, TSE – turbo spin-echo, cor – coronal, ax – axial, SPAIR – Spectral Attenuated Inversion Recovery, n/a – not applicable.

| | **T1w** | **T2w** | **T2w** | **PDw fs** |
|---|---|---|---|---|
| **Sequence Type** | 2D TSE | 2D TSE | 2D TSE | 3D TSE |
| **Orientation** | cor | cor | ax | cor |
| **Fat Saturation** | n/a | n/a | n/a | SPAIR |
| **Repetition Time [ms]** | 671 | 3283 | 3000 | 1200 |
| **Echo time [ms]** | 9 | 80 | 80 | 207 |
| **Turbo spin-echo factor** | 3 | 14 | 14 | 45 |
| **Field of view [mm]** | 160 x 160 | 160 x 160 | 160 x 160 | 170 x 170 |
| **Acquisition matrix** | 368 x 317 | 256 x 210 | 352 x 295 | 320 x 259 |
| **Reconstruction matrix** | 448 x 448 | 512 x 512 | 512 x 512 | 480 x 480 |
| **Scan percentage [%]** | 86 | 85 | 85 | 64 |
| **Flip angle [°]** | 90 | 90 | 90 | 90 |
| **Number of Signal Averages** | 1 | 1 | 1 | 1 |
| **Slices** | 30 | 40 | 30 | 229 |
| **Slice Thickness/ Gap [mm]** | 3.0 / 0.3 | 2.0 / 0.33 | 4.0 / 0 | 0.66 / 0.33 |
| **Duration [min:sec]** | 6:40 | 1:45 | 3:00 | 6:53 |

## Supplementary Figures

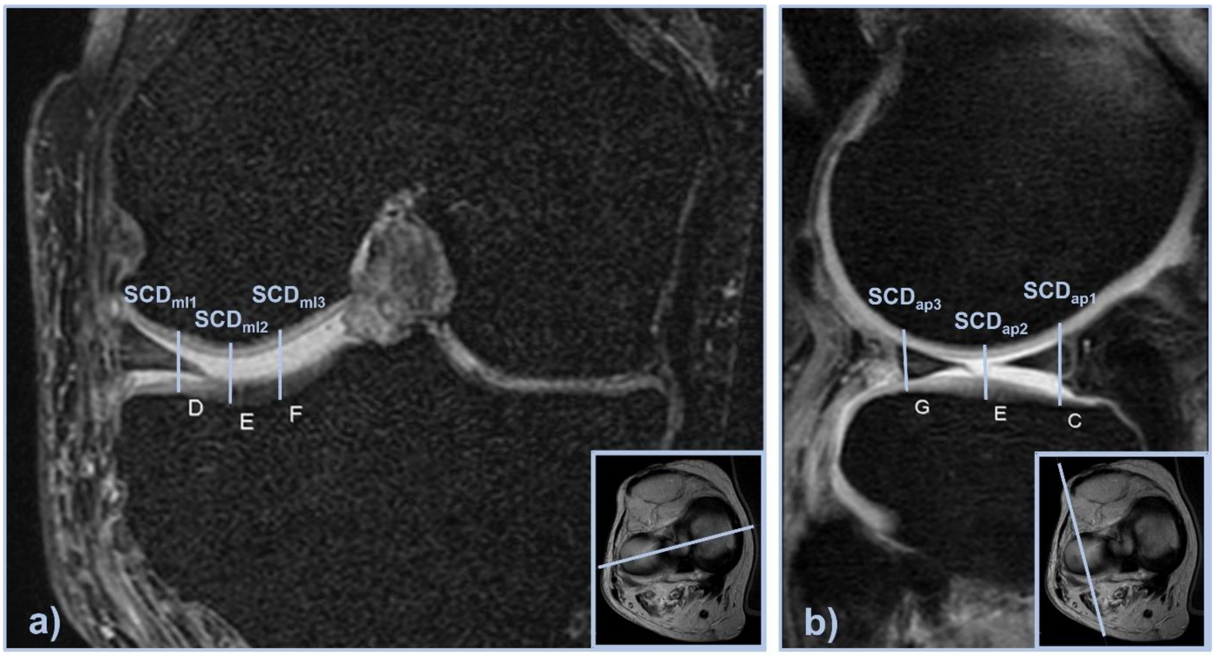


**Supplementary Figure 1:** Visualization of the manual 2D reference measurements**.** Lateral compartment opening was quantified by measuring the subchondral cortical distances (SCDs) at three locations in the mediolateral (a) and anteroposterior (b) dimension as indicated by the light blue vertical lines. Mid-coronal (a) and mid-sagittal (b, with respect to the lateral compartment) images of the 3D Proton Density-weighted sequence with fat saturation. Measurement locations are labelled $SCD_{ml1-3}$ and $SCD_{ap1-3}$. Slice orientation with respect to the axial orientation as indicated in the inset boxes.

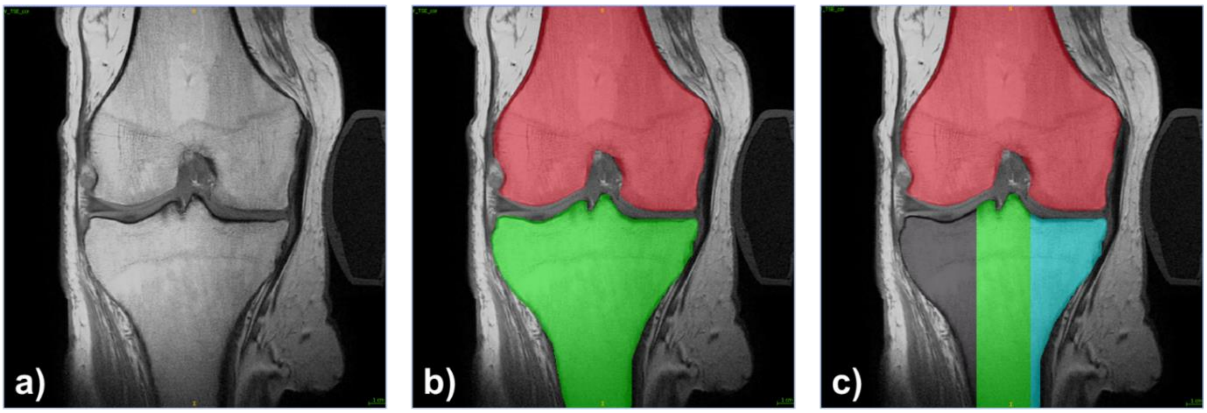


**Supplementary Figure 2**: Manual segmentation and labeling. Using the T1-weighted coronal images (a), the femur (red) and the tibia (green) (b) were manually segmented. The medial (light blue) and lateral (dark grey) compartments of the tibia were labeled separately (c), while the intercondylar eminence was spared (c).
